# Epitaxially grown BaM hexaferrite films having uniaxial axis in the film plane for self-biased devices

**DOI:** 10.1038/srep44193

**Published:** 2017-03-09

**Authors:** Xiaozhi Zhang, Siqin Meng, Dongsheng Song, Yao Zhang, Zhenxing Yue, Vincent G. Harris

**Affiliations:** 1 State Key Laboratory of New Ceramics and Fine Processing, School of Materials Science and Engineering, Tsinghua University, Beijing 100084, China; 2 National Center for Electron Microscopy in Beijing, Key Laboratory of Advanced Materials (MOE), School of Materials Science and Engineering, Tsinghua University, Beijing 100084, China; 3 Center for Microwave Magnetic Materials and Integrated Circuits, and the Department of Electrical and Computer Engineering, Northeastern University, Boston, Massachusetts 02115, USA

## Abstract

Barium hexaferrite (BaM) films with in-plane *c*-axis orientation are promising and technically important materials for self-biased magnetic microwave devices. In this work, highly oriented BaM films with different thickness and an in-plane easy axis (*c*-axis) of magnetization were grown on *a*-plane 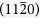 single-crystal sapphire substrates by direct current magnetron sputtering. A procedure involving seed layers, layer-by-layer annealing was adopted to reduce the substrate-induced strains and allow for the growth of thick (~3.44 μm) films. The epitaxial growth of the BaM film on sapphire was revealed by high-resolution transmission electron microscopy with dislocations being observed at the film-substrate interface. The orientation was also verified by X-ray diffraction and more notably, polarized Raman scattering. The magnetic properties and ferromagnetic resonant frequencies were experimentally characterized by a vibrating sample magnetometry and a frequency-swept ferromagnetic resonant flip-chip technique, respectively. The micron-thick BaM films exhibited a large remanence ratio of 0.92 along in-plane easy axis and a small one of 0.09 for the in-plane hard axis loop measurement. The FMR frequency was 50.3 GHz at zero field and reached 57.9 GHz under a magnetic field of 3 kOe, indicating that the epitaxial BaM films with strong self-biased behaviors have good electromagnetic properties in millimeter-wave range.

M-type barium hexaferrite BaFe_12_O_19_ (BaM) has attracted increasing attention for application in various microwave devices due to its large uniaxial magnetic anisotropy, relatively high permeability, and low conduction losses^1^,^2^,^3^,^4^,^5^,^6^,^7^,^8^. Hexaferrite films, especially *c*-axis aligned BaM films, are promising candidates for next-generation miniature planar high-frequency milllimeter wave ferrite devices^3^,^4^,^6^. Out-of-plane *c*-axis (OCA) oriented BaM films, with the magnetic moments perpendicular to the plane of the substrate, were first prepared in 1981 by M. Naoe *et al*. to be used as perpendicular magnetic recording media to replace Co-Cr thin films^9^,^10^. Up to now, OCA BaM films have been extensively studied and successfully fabricated by different methods, such as molecular beam epitaxy (MBE)^11^,^12^, liquid phase epitaxy (LPE)^13^, alternating target laser ablation deposition (ARLAD)^14^,^15^, pulsed laser deposition (PLD)^16^,^17^,^18^, radio frequency (RF) magnetron sputtering^19^,^20^,^21^, spin-coating^22^,^23^,^24^ and so on. Utilizing an external magnetic biasing field to induce the alignment of *c*-axes of the BaM particles, instead of the epitaxial growth methods mentioned above, has also been adopted and includes screen printing (SP)^25^,^26^ and electrophoretic deposition (EPD)^27^, to name a few. The (0001) textured films, however, usually have small remanent magnetization (*M*_r_) and are hard to be made fully self-biasing due to their large shape-demagnetizing field for this particular geometry^28^. Self-biasing is an important property that eliminates the need of a large external magnet, which provides the needed biasing field for magnetic microwave devices. This is an essential step in making these devices and related components lighter and smaller^5^.

In principle, the realization of a high remanence ratio *M*_r_/*M*_s_ value for in-plane *c*-axis oriented (IPCA) BaM films is much easier as compared to the (0001) textured ones, since IPCA films have a near-zero shape-demagnetizing factor in the film plane and self-demagnetization effects are minimized^4^,^28^,^29^,^30^. Another basic advantage of IPCA BaM films lies in their higher ferromagnetic resonance (FMR) frequency, which reaches ~50 GHz at zero external field, about 45% higher than that of OCA ones^29^. However, unlike OCA BaM films, which have been fabricated on various substrates, like Al_2_O_3_ (0001)^16^,^20^,^24^, MgO (111)^31^, SiC (0001)^12^,^17^,^18^, Si (111)^19^, GaN (0001)^32^ and Gd_3_Ga_5_O_12_ (GGG) (111)^13^, successful fabrication of the IPCA BaM films has always been a challenge. It was not until the year 1992 that Hlyton *et al*. reported the *c*-axis aligned BaM films with in-plane anisotropy for the first time by reactive magnetron sputtering^33^. Progress for IPCA BaM films has been limited after that, owing to the limited selection of preparation methods and substrates, mainly *a*- and *m*-plane sapphire (Al_2_O_3_). Some recent successes regarding the fabrication of highly textured IPCA BaM films have been reported using PLD^28^,^34^ or a hybrid process that involved PLD and LPE^29^,^30^,^35^,^36^. LPE remains the primary technique for processing near single-crystal quality BaM films with large thickness (up to 80 μm for IPCA oriented ones) and low microwave loss. The low-loss property, however, comes together with a low coercivity, which typically corresponding to a low remanence ratio^4^,^5^,^6^. In other words, although possessing a very high crystal quality, the IPCA BaM film fabricated by LPE always has a low remanence ratio, which ranges from ~0.84 at 7 μm to ~0.33 at 20 μm, and drops to a near-zero value with even larger thickness^29^,^35^,^36^,^37^. What’s more, LPE involves a complicated liquid phase control, and requires expensive equipment, which hinders its application in industrial production. The industrial production cost is also very high for PLD technique, whose deposition rate is limited by the laser power. In addition, both LPE and PLD are restricted to relatively small size substrates.

Industrial-scale film processing technique that requires no high-end equipments, e.g., magnetron sputtering, are long known as a crude method for epitaxial growth. Specifically, direct current (DC) magnetron sputtering has long been used to sputter metal materials, while oxide ceramics have always been prepared by RF magnetron sputtering, which is subject to low sputtering rate compared with DC sputtering. In our previous studies, however, well aligned in-plane *c*-axis oriented BaM thin films (∼120 nm in thickness) were successfully prepared using DC magnetron sputtering^38^,^39^. In the present work, using seed layers and a layer-by-layer annealing procedure, we obtained 3.44-μm-thick epitaxially grown BaM films that hold promise for various microwave applications. Furthermore, the microstructure and epitaxial orientation relationship between BaM and sapphire were comprehensively investigated based on transmission electron microscopy (TEM) that has seldom been reported for this system and Raman analysis that is efficient in probing structures and strains in small regions^40^,^41^,^42^,^43^. Using a flip-chip technique^44^, the frequency-swept ferromagnetic resonant (FMR) measurements were carried out. The increasing of FMR frequency of the BaM film with increasing external magnetic field was observed, with the FMR frequency at zero field extrapolated to be 50.3 GHz, implying their potential in the millimeter-wave magnetic film device applications.

## Results and Discussion

**Figure 1:**
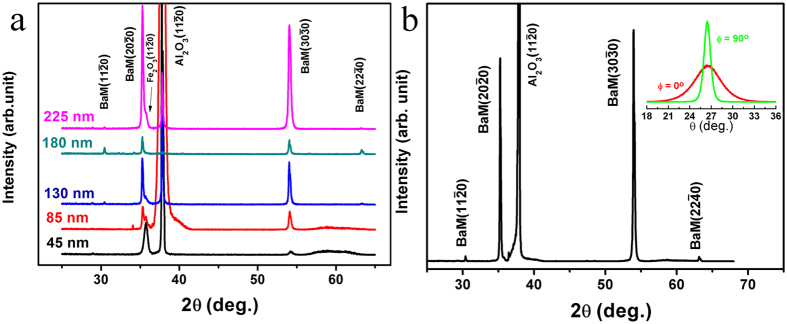
X-ray diffraction patterns of (**a**) monolayer BaM films with different thickness, and (**b**) 3.44-μm-thick film sample prepared by layer-by-layer annealing procedure. Inset in (**b**) is the rocking curves of 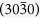 peak for the 3.44-μm-thick film along two different directions, where Φ is the angle between *c*-axis of BaM and the plane formed by the incident and reflected X-rays.

Figure 1 illustrates the XRD patterns of the BaM films with different thickness. All the samples, except the thinnest one, show an obvious in-plane *c*-axis orientation with two main peaks being indexed to the 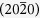 and 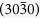 planes of BaM. The *α*-Fe_2_O_3_ phase is also detected in the films owing to its higher thermodynamic stability than BaM and tendency of persisting during BaM fabrication^38^,^39^,^45^. Our previous studies have shown that *α*-Fe_2_O_3_ phase plays a significant role in promoting the in-plane *c*-axis oriented growth of the BaM films on sapphire substrates^38^,^39^, and acts as a temporary template during the orientation growth of the BaM crystallites^20^. Two small peaks corresponding to 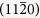 and 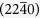 crystallographic planes of BaM also appear in the XRD patterns. Both 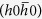 and 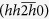 peaks show an in-plane *c*-axis orientation of BaM, with their *c*-axes being parallel to that of the Al_2_O_3_ substrate, but with a 30° rotation around *c*-axis between them. In general, 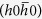 peaks (*m*-plane) of BaM film would appear on *a*-cut plane sapphire substrate and 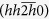 peaks (*a*-plane) of BaM on *m*-cut sapphire, according to the orientation relationship between BaM and sapphire. It is noticed that there is no obvious shift of the peaks with thickness whose effect will be further clarified by Raman spectra as will be discussed later.

A lattice shrinkage of the films was suggested when comparing the peak positions of 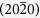 and 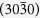 with the powder samples (PDF 39-1433) due to the compressive stress^35^ that originates from the lattice mismatch between the film and the substrate. This mismatch also leads to difficulty in preparing a film sample thicker than ~250 nm, and this is the reason why the layer-by-layer annealing method was employed to get micron thick BaM films. Inset in Fig. 1(b) shows the rocking curves of the 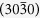 peak of the 3.44-μm-thick BaM film that was measured along two mutually orthogonal directions: Φ = 0° and Φ = 90°. Compared with Φ = 90°, the Φ = 0° peak is noticeably wider, which is caused by the small grain size along *c*-axis^39^.

**Figure 2:**
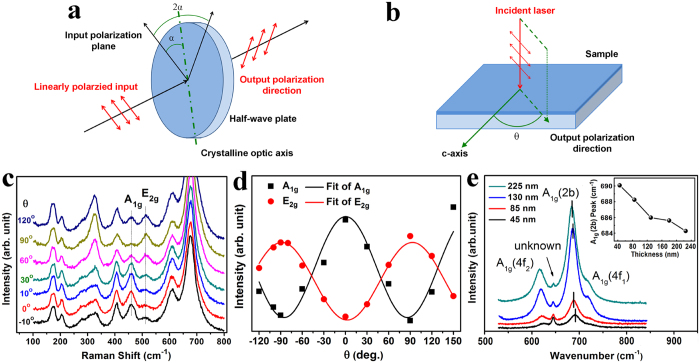
(**a**) Schematic diagram showing the polarization direction of the incident laser is modulated by a half-wave plate. No polarization plate was used here due to the difficulty in making the polarization plate to rotate synchronously with the half-wave plate. (**b**) Schematic diagram showing the angle *θ* between output polarization direction and *c*-axis of the BaM film. (**c**) Raman spectra of the 3.44-μm-thick BaM film at different *θ* values. (**d**) Dependence of the intensities of *A*_1g_ and *E*_2g_ peaks on angle *θ* derived from (**c**). (**e**) Raman spectra of the monolayer BaM films with different thickness in high wavenumber domain, and the inset is the dependence of *A*_1g_(2*b*) peak position on film thickness.

It is also noticed that the effect of thickness is not clearly reflected in the XRD patterns as shown in Fig. 1, so additional characterization is needed to study thickness-induced structural variation. It is known that Raman spectroscopy is much more sensitive for probing such small difference in film thickness, which was used here to further illustrate the orientated growth of BaM films and particularly the influence of thickness. A half-wave plate was employed to modulate the polarization direction of the incident laser as shown in Fig. 2(a), and the geometrical relationship between the film sample and the Raman spectrometer is schematically depicted in Fig. 2(b). In this work, the 3.44-μm-thick sample was held fixed in space and the *c*-axis of BaM film was aligned perpendicular to the direction of the incident laser. Raman spectra were measured at different *θ* vaules, and all Raman scattering bands shown in Fig. 2(c) were attributed to the characteristic peaks of BaM^40^. Specifically, the bands at about 460 and 515 cm^−1^ were indexed to *A*_1g_ and *E*_2g_ vibrations, respectively^40^,^42^. It is found that the peak intensity of *A*_1g_ reaches its zenith at *θ* = 0°, while *E*_2g_ at *θ* = 90°. In other words, when 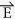, the electric field vector of the incident laser, aligns parallel to *c*-axis of BaM, the intensity of *A*_1g_ peak reaches its maximum value; and when 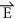 is perpendicular to *c*-axis, the intensity of *E*_2g_ reaches its highest value. The sensibility of the intensities of *A*_1g_ and *E*_2g_ peaks are consistent with the in-plane anisotropy of the BaM film and in agreement with XRD results.

To quantitatively analyze the Raman intensity in relation to *θ*, we fitted the two peaks and obtained the integrated intensity after subtracting a linear baseline. The experimentally determined angular dependence of the intensities of *A*_1g_ and *E*_2g_ peaks for the 3.44-μm-thick BaM film is presented in Fig. 2(d). We can see that the intensities of both 460 cm^−1^ and 515 cm^−1^ peaks vary gradually with a periodicity of π. The monolayer BaM films also show the similar phenomenon, and such behavior in peak intensity is markedly different from XRD, where even a small deviation in orientation can cause diffracted intensities to drop dramatically. Similar phenomenon of the periodically changed Raman peak intensity has never been observed on BaM films with their *c*-axis randomly distributed in the film plane, even though the XRD patterns also show strong in-plane oriented peak intensities. This part of work will be soon reported. Raman spectra of the BaM films with different thickness in high wavenumber domain are presented in Fig. 2(e). The bands at about 615, 685, and 720 cm^−1^ are attributed to *A*_1g_ vibrations at the octahedral (4*f*_2_), bipyramidal (2*b*), and tetrahedral (4*f*_1_) polyhedra sites, respectively^40^,^46^. The denoted unknown peaks between *A*_1g_(4*f*_2_) and *A*_1g_(2*b*) are not found in the 3.44-μm-thick BaM films, and probably come from the interface region between BaM thin films and sapphire substrates. Both *A*_1g_(4*f*_2_) and *A*_1g_(2*b*) (shown in the inset) bands exhibit an obvious redshift with the increasing film thickness, suggesting a release of the strain inside BaM with the increasing film thickness^47^,^48^.

**Figure 3:**
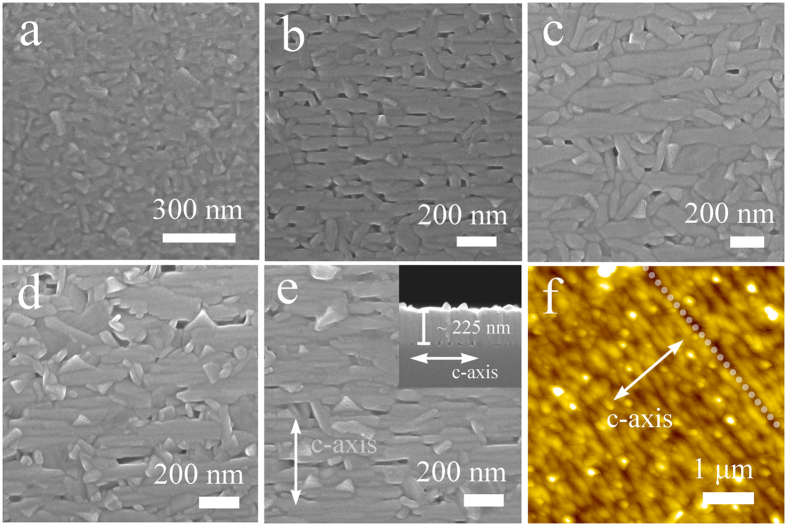
SEM surface images of the monolayer BaM films with different thickness. (**a**) 45 nm, (**b**) 85 nm, (**c**) 130 nm, (**d**) 180 nm, and (**e**) 225 nm. Inset in (**e**) shows the cross-sectional SEM image of the 225-nm-thick BaM film. (**f**) AFM surface microstructure of the 3.44-μm-thick BaM film. The arrows in the images indicate the *c*-axis of BaM.

Microstructures of the BaM films with different thickness are presented in Fig. 3. All the samples, except the thinnest one, are composed of parallel aligned grains, indicating in-plane orientation of *c*-axis^38^,^39^. The surface morphology of the 45-nm-thick sample, as shown in Fig. 3(a), does not show obvious parallel aligned grains, in accordance with the XRD results in Fig. 1(a). The cross-sectional SEM image of the 225-nm-thick film shown in inset of Fig. 3(e) reveals a clearly layered structure along *c*-axis. AFM surface image of the 3.44-μm-thick film given in Fig. 3(f) demonstrates a reasonably smooth surface and also some fine lines along the direction perpendicular to *c*-axis of the BaM film, suggesting good orientation of *c*-axis of the BaM film^30^,^34^.

**Figure 4:**
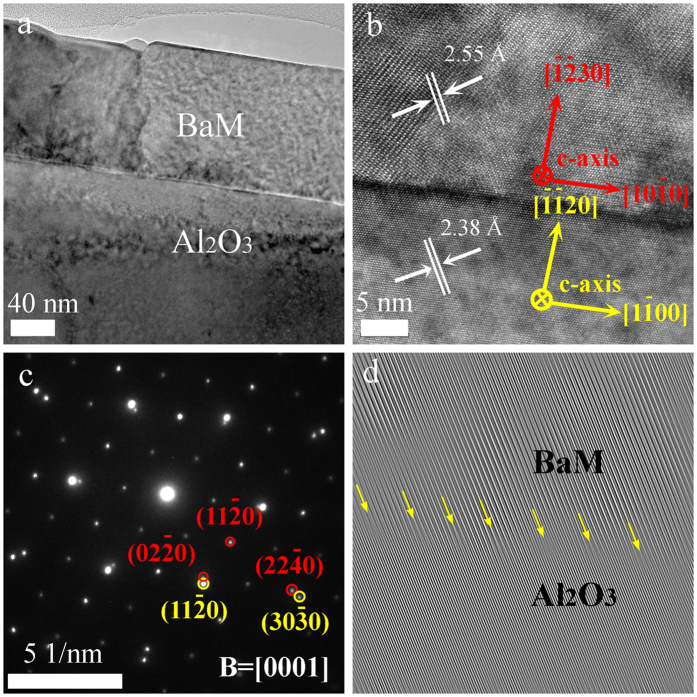
(**a**) Cross-sectional low-magnificaiton BF-TEM image of 130-nm-thick BaM/Al_2_O_3_ interface. (**b**) High-resolution TEM image showing the epitaxial growth of BaM film on sapphire. (**c**) Selected area electron diffraction patterns along the [0001] direction from the film-substrate interface region, where spots circled in red and yellow belong to BaM film and Al_2_O_3_ substrate, respectively. (**d**) The filtered FFT patterns of (**b**) showing the dislocations at the film-substrate interface.

The epitaxial growth of BaM film (130-nm-thick one as an example) on sapphire was further confirmed by TEM as shown in Fig. 4. The bright field (BF) TEM image shown in Fig. 4(a) exhibits a uniformly grown BaM film on Al_2_O_3_, with the grains extending across the full thickness of the film. The high resolution TEM (HRTEM) image in Fig. 4(b) reveals the atomic structure at the interface between BaM and Al_2_O_3_ on (0001) planes. Measured lattice distances read 2.38 Å and 2.55 Å for Al_2_O_3_ (*d*_11-20_) substrate and BaM (*d*_20-20_) film, respectively. It gives a misfit parameter of about 7% between the two structures on (0001), the oxygen close-packing planes. The selected area diffraction (SAD) pattern in Fig. 4(c) from the BaM/Al_2_O_3_ interface area reveals the orientation character: BaM[0001]//Al_2_O_3_[0001] and BaM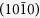//Al_2_O_3_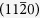, and both BaM and sapphire show a 6-fold symmetry. In addition, there exists a 30° rotation around [0001] direction between BaM and sapphire, which is in accordance with the XRD patterns shown in Fig. 1(a). The splitting of the diffraction spots shown in Fig. 4(c) confirms the crystal mismatch between BaM and sapphire seen in Fig. 4(b). To further understand this phenomenon, we use the fast Fourier transform (FFT) to identify the interfacial dislocations in Fig. 4(d) as indicated by the arrows. Unsurprisingly, the dislocations periodically appear at the interface every dozen of lattice fringes, or about every 8 unit cells, indicating a misfit parameter of ~7% between BaM and sapphire in the (0001) planes. The formation of misfit dislocations at the interface by strain relaxation infers the epitaxial growth of the film on substrate^49^,^50^,^51^. The strains initiated at the interface will influence the magnetic properties of the entire film, as will be shown by the following VSM results. Nevertheless, using a layer-by-layer annealing procedure, the stain got considerably relieved by forming new interfaces between each layer, especially for the initially deposited 130-nm-thick seed layer.

**Figure 5:**
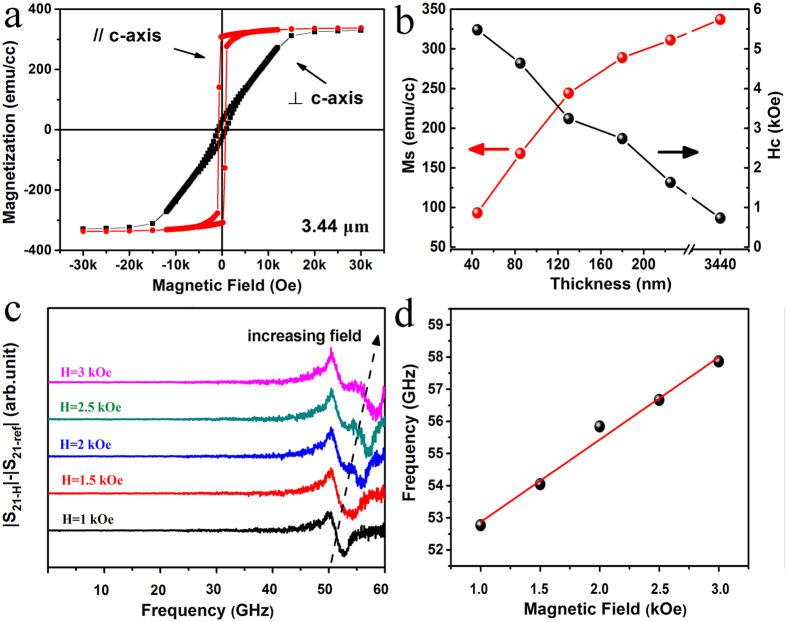
(**a**) Magnetic hysteresis curves of the 3.44-μm-thick BaM films, with the loops measured with the magnetic field along the in-plane easy and hard axes of BaM, respectively. (**b**) Dependence of saturation magnetization and coercivity on film thickness along the in-plane easy axis. (**c**) Transmission coefficient *S*_21_ (with the background subtracted off) frequency-domain spectrums of the 3.44-μm-thick BaM film tested under various DC magnetic field. The sample was applied with its *c*-axis along the direction of the external magnetic field. The background data is obtained by measuring *S*_21_ without an applied magnetic field. (**d**) Dependence of ferromagnetic resonant frequency on applied magnetic field, in accordance to data from (**c**). The dots are the peak values of the absorption peaks at different fields, and the solid line is a linear fitting to the data.

Magnetic property measurements of the BaM films with different thickness were conducted along in-plane easy ([0001]) and hard (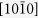) axes using vibrating sample magnetometry. The magnetic data, including saturation magnetization (*M*_s_), remanent magnetization (*M*_r_), coercivity (*H*_c_) and effective anisotropy field (*H*_A_) are listed in Table 1 and illustrated in Fig. 5(a) and Supplementary Information S1. It can be seen that all the BaM films with different thickness exhibit obvious in-plane anisotropy. The *M*_s_ value is only 90 emu/cc for the 45-nm-thick film sample due to the existence of the non-magnetic *α*-Fe_2_O_3_ phase^33^. With the increasing thickness, the amount of *α*-Fe_2_O_3_ phase decreases, and *M*_s_ value of BaM increases accordingly as indicated in Fig. 5(b). For the 3.44-μm-thick film sample, where no *α*-Fe_2_O_3_ phase was detected by XRD, *M*_s_ is 337 emu/cc and close to (~89%) the value reported for bulk and crystal materials^52^. The fact that *M*_s_ of the 3.44-μm-thick BaM film is not 100% of the theoretical value of BaM (380 emu/cc) was tentatively attributed to Al diffusion from the sapphire substrates, as confirmed by both the TEM-EDS line scan (Supplementary Information S2) and XPS results (Supplementary Information S3). The hysteresis loops along in-plane hard axis are almost linear and closed (coercivity is small) for the 225-nm-thick and 3.44-μm-thick BaM films, indicating that the easy axes are well aligned in the film plane with small *c*-axis dispersion^39^,^45^,^53^. Nevertheless, the *H*_c_ vaule increases rapidly with reducing thickness as shown in Fig. 5(b). It suggests that the strains are initiated at the interface and have an influence on the magnetic properties of the entire film^53^. In addition, the in-plane *c*-axis aligned BaM films, only except the 45-nm-thick ones, have large remanent magnetization to saturation magnetization (*M*_r_/*M*_s_) ratio of larger than 0.85 along *c*-axis as shown in Table 1. This large ratio is comparable with the reported films prepared by radio frequency (RF) magnetron sputtering and pulsed laser deposition (PLD) techniques^34^,^45^.

Transmission coefficient *S*_21_ for the 3.44-μm-thick BaM film was measured under various DC magnetic field in a broadband frequency-swept FMR spectrometer system, and illustrated in Fig. 5(c). Due to the lower signal level, the FMR spectra for the thinner films were not obtained in our measurement system. Thus, the FMR frequencies were calculated via Kittel equation, and the data are included in Table 1. It can be seen that all films with different thickness, except the 45-nm-thick film, show zero-field FMR frequencies higher than 50 GHz, which can be considered the result of the high in-plane *c*-axis orientation. The little influence of film thickness on the FMR frequency can be also observed from Table 1 for the films with thickness ranging from 85 nm to 3.44 μm. The relative lower FMR frequency of the 45-nm-thick film may be caused by the poorer orientation as discussed above. From Fig. 5(c), the absorption peaks that positioned downward originate from FMR, while the ones that positioned upward at ~50 GHz are caused by the subtraction of the background at zero fields. This background is composed of two main parts. One is the zero-field FMR response of the 3.44-μm-thick film sample, which was extrapolated to be ~50.3 GHz according to Fig. 5(d). This value is well consistent with the calculated one (50.9 GHz, Table 1) according to Kittel equation. The second part comes from the background loss and noise caused by transmission lines and the fixture, which are also nearly static and can be easily distinguished. The FMR frequency of BaM increases with the increasing magnetic field, and Fig. 5(d) represents a linear dependence of the FMR frequency on the applied magnetic field strength. The full width half maximum (FWHM) linewidth Δƒ is ~2 GHz, using a Lorentzian fit on the data collected at 2.5 kOe, as the green line shows in Fig. 5(c). The more commonly used field linewidth Δ*H* is calculated to be ~0.78 kOe by dividing Δƒ by γ^44^, the gyromagnetic ratio, which corresponds to the slope in Fig. 5(d) and is ~2.56 GHz/kOe. The Δ*H* value is comparative to that micron-thick in-plane *c*-axis oriented BaM films (~0.2–0.4 kOe) grown by PLD technique^30^,^34^. The larger FWHM linewidth of the present films might be caused by the imperfect crystalline quality of the layer-by-layer annealed film samples, which can be improved by further optimization of deposition processing, especially the annealing procedure. Anyway, the above results suggest that the epitaxial BaM film with a relatively large thickness prepared by DC magnetron sputtering is a promising candidate for application in self-biased microwave and millimeter-wave devices.

## Conclusion

In-plane *c*-axis oriented Ba-hexaferrite (BaM) films with different thickness were deposited on *a*-plane 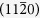 sapphire (Al_2_O_3_) substrates using DC magnetron sputtering followed by an *ex-situ* annealing process. Seed layer and a layer-by-layer annealing procedure were adopted to fabricate micron-thick BaM films, with the seed layer acting as a buffer layer to relieve the strains and induce further orientated growth of the thick film. The epitaxial growth of BaM on sapphire was confirmed by high resolution transmission electron microscopy, polarized Raman spectra, as well as X-ray diffraction results. Dislocations were observed across the film-substrate interface and were expected to relieve the strain inside BaM and facilitate the epitaxial growth of the films. All the BaM films exhibit obvious in-plane uniaxial anisotropy, holding great promise for application in self-biased millimeter-wave devices. The present work should be helpful for preparing highly orientated oxide films and understanding their epitaxial structures as well as the relationship between structure and properties.

## Methods

### Film Deposition

BaFe_12_O_19_ films were grown on polished 5 × 5 mm^2^ sapphire (Al_2_O_3_) substrates using a DC magnetron sputtering technique^38^,^39^. The BaM hexagonal structure, with *m*-plane alignment, grew epitaxially on *a*-plane of sapphire substrates. The deposition condition included sputtering gases of Ar-7% O_2_, DC sputtering power of 100 W, and substrate temperature set at room temperature. Different deposition duration time was set as 5, 10, 20, 30, and 40 min to get BaM films with different thickness of about 45, 85, 130, 180, and 225 nm, respectively. The as-deposited amorphous films were subsequently annealed at 850 °C for 30 min in air. It was found that 225 nm was the largest thickness that a monolayer BaM film could reach with a good repeatability. The thickness limit is probably caused by the strains in the film, which mainly come from the large thermal and lattice mismatch between BaM and sapphire^29^,^33^,^35^,^53^,^54^. To solve this problem and obtain micron-thick BaM films, a layer-by-layer annealing procedure was adopted, taking the 130-nm-thick film as a seed layer to reduce the strain between film and substrate and induce the orientated growth of the entire micron-thick BaM film as well. In the following 6-layer deposition, the deposition time for each layer was set to 90 min, annealing time 10 min, while the annealing temperature varied for different layers. The first and second layers were annealed at 840 °C, the third and fourth layers at 830 °C, and the fifth and sixth at 820 °C, respectively. The decreasing annealing temperature procedure was designed to prevent excessive grain growth.

### Structural Characterization

Phase and crystallographic orientation were studied by *θ*–*2θ* X-ray diffraction (XRD) using a Rigaku Smartlab diffractometer (Cu *K*_α_ radiation). Surface morphology of the films was observed using scanning electron microscope (SEM; MERLIN VP Compact, Carl Zeiss, Germany) and atomic force microscopy (AFM; SPI 4000, Seiko, Japan). Film thickness was estimated on the cross-sectional microscope sample using SEM. X-ray photoelectron spectra (XPS) were recorded using a Thermo Fisher Escalab 250Xi spectroscopy with Al *K*_α_ X-ray source (E = 1486.6 eV). Atomic structure and chemical composition at BaM-sapphire interface were characterized by transmission electron microscopy (TEM) on the FEI Tecnai G^2^ F20 microscope attached with energy dispersive X-ray spectrometer (EDS). Raman scattering spectra were recorded in a backscattering configuration using a Raman spectrometer (RS; LabRAM HR Evolution, HORIBA Scientific, Japan). A 633 nm laser was used as the excitation source with ~1 mW power incident upon the surface of BaM film.

### Static Magnetic and Microwave Properties Measurements

Static magnetic measurements were performed at room temperature using a superconducting quantum interference device (SQUID-VSM, Quantum Design, USA), with the magnetic hysteresis loops measured along the in-plane easy and hard axes to a maximum field of 3 Tesla. Microwave property measurements were carried out using a broadband frequency-swept FMR spectrometer with a flip-chip configuration^44^,^55^, in which the BaM film was positioned face-down on a coplanar waveguide. The transmission responses of the coplanar waveguide were detected using a vector network analyzer (VNA) Agilent E8361 C at varied direct current (DC) magnetic field, with the in-plane easy axis (*c*-axis) of the film sample applied parallel to the applied DC magnetic field. The transmission coefficient *S*_21_ was measured at a constant field with the background subtraction positioned off without an applied magnetic field. This test method was adopted to obtain high-quality data for ferromagnetic resonant (FMR) frequency and minimize interference from other sources of absorption.

### FMR frequency calculation from Kittle equation

The FMR frequency at zero field shown in Table 1 was calculated using Kittel equation^56^,^57^.


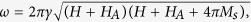


where *H* is the applied magnetic field, and γ is the gyromagnetic ratio, which can be set as 2.8 MHz/Oe^29^, while the values of effective anisotropy field *H*_A_ and saturation introduction 4π*M*_s_ are taken from Table 1.

## Additional Information

**How to cite this article:** Zhang, X. *et al*. Epitaxially grown BaM hexaferrite films having uniaxial axis in the film plane for self-biased devices. *Sci. Rep.* **7**, 44193; doi: 10.1038/srep44193 (2017).

**Publisher's note:** Springer Nature remains neutral with regard to jurisdictional claims in published maps and institutional affiliations.

## Supplementary Material

Supplementary Information

## Figures and Tables

**Table 1:** Magnetic properties of the BaM films grown on *a*-cut (11
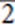
0) sapphire substrates.

Film thickness	In-plane hard axis				In-plane easy axis				*H*_A_(kOe)	FMR frequency(GHz)
	*M*_s_ (emu/cc)	*M*_r_ (emu/cc)	*M*_r_/*M*_s_	*H*_c_ (Oe)	*M*_s_ (emu/cc)	*M*_r_ (emu/cc)	*M*_r_/*M*_s_	*H*_c_ (Oe)		
45 nm	90	27.5	0.31	3358	93	64.2	0.69	5480	16.1	46.7
85 nm	160	33.5	0.21	2590	168	142.4	0.85	4639	17.3	51.3
130 nm	253	59.8	0.24	3189	263	225.2	0.86	3239	17.3	52.9
180 nm	276	67.9	0.25	2876	289	248.7	0.86	2736	16.9	52.2
225 nm	282	13.7	0.05	346	311	281.6	0.91	1630	17.3	53.6
3.44 μm	329	28.5	0.09	905	337	308.8	0.92	730	16.2	50.9

The applied field H is parallel to the substrate surface for both the easy and hard axis loops measurements. The diamagnetic background of the sapphire substrate has been subtracted for all the samples to exclusively reveal the magnetic properties of the films. The FMR frequency values under zero field for all the samples were calculated by Kittel equation.

## References

[b1] Pullar R. C. Hexagonal ferrites: A review of the synthesis, properties and applications of hexaferrite ceramics. Prog. Mater Sci. 57, 1191–1334 (2012).

[b2] Özgür Ü., Alivov Y. & Morkoç H. Microwave ferrites, part 1: fundamental properties. J. Mater. Sci.: Mater. Electron. 20, 789–834 (2009).

[b3] Adam J. D., Davis L. E., Dionne G. F., Schloemann E. F. & Stitzer S. N. Ferrite devices and materials. *IEEE Trans*. Microwave Theory Tech. 50, 721–737 (2002).

[b4] Harris V. G. Modern microwave ferrites. IEEE Trans. Magn. 48, 1075–1104 (2012).

[b5] Harris V. G. . Ba-hexaferrite films for next generation microwave devices. J. Appl. Phys. 99, 08M911 (2006).

[b6] Harris V. G. . Recent advances in processing and applications of microwave ferrites. J. Magn. Magn. Mater. 321, 2035–2047 (2009).

[b7] Su Z., Bennett S., Hu B., Chen Y. & Harris V. G. Magnetic and microwave properties of U-type hexaferrite films with high remanence and low ferromagnetic resonance linewidth. J. Appl. Phys. 115, 17A504 (2014).

[b8] Liu J., Zeng Y., Guo C., Zhang W. & Yang X. One-step synthesis of barium hexaferrite nano-powders via microwave-assisted sol–gel auto-combustion. J. Eur. Ceram. Soc. 30, 993–997 (2010).

[b9] Naoe M., Hasunuma S., Hoshi Y. & Yamanaka S. Preparation of barium ferrite films with perpendicular magnetic anisotropy by DC sputtering. IEEE Trans. Magn. 17, 3184–3186 (1981).

[b10] Matsuoka M., Naoe M. & Hoshi Y. Ba–ferrite thin‐film disk for perpendicular magnetic recording. J. Appl. Phys. 57, 4040–4042 (1985).

[b11] Liu H. . Epitaxial relationship of MBE grown barium hexaferrite (0001) films on sapphire (0001). J. Cryst. Growth 312, 671–675 (2010).

[b12] Ohodnicki P. R. . Correlation between texture, anisotropy, and vector magnetization processes investigated by two-dimensional vector vibrating sample magnetometry in BaO(Fe_2_O_3_)_6_ thin film. J. Appl. Phys. 103, 07E514 (2008).

[b13] Yoon S. D. & Vittoria C. Thick M-type barium hexaferrite films grown on garnet substrates. J. Appl. Phys. 96, 2131–2135 (2004).

[b14] Geiler A. L. . Atomic scale design and control of cation distribution in hexagonal ferrites. Phys. Rev. Lett. 101, 067201 (2008). 18764494 10.1103/PhysRevLett.101.067201

[b15] Mohebbi M., Ebnabbasi K. & Vittoria C. *In-situ* deposition of *c*-axis oriented barium ferrite films for microwave applications. IEEE Trans. Magn. 49, 4207–4209 (2013).

[b16] Carosella C. A. . Pulsed laser deposition of epitaxial BaFe_12_O_19_ thin films. J. Appl. Phys. 71, 5107–5110 (1992).

[b17] Chen Z. . Growth of Ba-hexaferrite films on single crystal 6-H SiC. J. Magn. Magn. Mater. 301, 166–170 (2006).

[b18] Cai Z., Chen Z., Goodrich T. L., Harris V. G. & Ziemer K. S. Chemical and structural characterization of barium hexaferrite films deposited on 6H-SiC with and without MgO/BaM interwoven layers. J. Cryst. Growth 307, 321–327 (2007).

[b19] Zhang L., Su X. D., Chen Y., Li Q. F. & Harris V. G. Radio-frequency magnetron sputter-deposited barium hexaferrite films on Pt-coated Si substrates suitable for microwave applications. Scripta Mater. 63, 492–495 (2010).

[b20] Cho T. S., Je J. H. & Noh D. Y. Formation of crystalline Ba-ferrite phase from *α*-Fe_2_O_3_ phase in amorphous precursor. Appl. Phys. Lett. 76, 303–305 (2000).

[b21] Gee S. H., Hong Y. K., Erickson D. W., Tanaka T. & Park M. H. *Ex situ* annealing method for *c*-axis oriented barium ferrite thick films. J. Appl. Phys. 93, 7507–7509 (2003).

[b22] Meng S., Yue Z., Zhang X. & Li L. Quasi-epitaxial barium hexaferrite thin films prepared by a topotactic reactive diffusion process. Appl. Surf. Sci. 290, 340–345 (2014).

[b23] Harward I. . Physical properties of Al doped Ba hexagonal ferrite thin films. J. Appl. Phys. 113, 043903 (2013).

[b24] Meng S., Yue Z. & Li L. Effect of ethylene glycol on the orientation and magnetic properties of barium ferrite thin films derived by chemical solution deposition. J. Magn. Magn. Mater. 354, 290–294 (2014).

[b25] Chen Y. . Oriented barium hexaferrite thick films with narrow ferromagnetic resonance linewidth. Appl. Phys. Lett. 88, 062516 (2006).

[b26] Chen Y. . Screen printed thick self-biased, low-loss, barium hexaferrite films by hot-press sintering. J. Appl. Phys. 100, 43907 (2006).

[b27] Lisjak D. & Ovtar S. The alignment of barium ferrite nanoparticles from their suspensions in electric and magnetic fields. J. Phys. Chem. B 117, 1644–1650 (2013). 22834411 10.1021/jp305256t

[b28] Yoon S. D., Vittoria C. & Oliver S. A. Magnetization behavior of scandium-substituted barium hexaferrite films having uniaxial axis in the film plane. J. Magn. Magn. Mater. 265, 130–137 (2003).

[b29] Song Y.-Y., Das J., Wang Z., Tong W. & Patton C. E. In-plane *c*-axis oriented barium ferrite films with self-bias and low microwave loss. Appl. Phys. Lett. 93, 172503 (2008).

[b30] Song Y.-Y., Sun Y., Lu L., Bevivino J. & Wu M. Self-biased planar millimeter wave notch filters based on magnetostatic wave excitation in barium hexagonal ferrite thin films. Appl. Phys. Lett. 97, 173502 (2010).

[b31] Hu B. . Magnetocrystalline anisotropy and FMR linewidth of Zr and Zn-doped Ba-hexaferrite films grown on MgO (111). IEEE Trans. Magn. 49, 4234–4237 (2013).

[b32] Ohodnicki P. R. . Magnetic anisotropy and crystalline texture in BaO(Fe_2_O_3_)_6_ thin films deposited on GaN∕Al_2_O_3_. J. Appl. Phys. 101, 09M521 (2007).

[b33] Hylton T. L., Parker M. A. & Howard J. K. Preparation and magnetic properties of epitaxial barium ferrite thin films on sapphire with in-plane, uniaxial anisotropy. Appl. Phys. Lett. 61, 867–869 (1992).

[b34] Li P. . Generation of pure spin currents via spin Seebeck effect in self-biased hexagonal ferrite thin films. Appl. Phys. Lett. 105, 242412 (2014).

[b35] Yoon S. D. & Vittoria C. Microwave and magnetic properties of barium hexaferrite films having the *c*-axis in the film plane by liquid phase epitaxy technique. J. Appl. Phys. 93, 8597–8599 (2003).

[b36] Wise A. T. . M-type barium hexaferrite synthesis and characterization for phase shifter applications. J. Appl. Phys. 109, 07E535 (2011)

[b37] Yoon S. D. & Vittoria C. Preparation of high-quality hexaferrite thick films by an improved liquid phase epitaxy deposition technique. IEEE Trans. Magn. 39, 3163–3165 (2003).

[b38] Zhang X., Yue Z., Meng S. & Yuan L. Magnetic properties of in-plane oriented barium hexaferrite thin films prepared by direct current magnetron sputtering. J. Appl. Phys. 116, 243909 (2014).

[b39] Zhang X., Yue Z. & Li L. Orientation growth and magnetic properties of BaM hexaferrite films deposited by direct current magnetron sputtering. J. Am. Ceram. Soc. 99, 860–865 (2016).

[b40] Kreisel J., Lucazeau G. & Vincent H. Raman spectra and vibrational analysis of BaFe_12_O_19_ hexagonal ferrite. J. Solid State Chem. 137, 127–137 (1998).

[b41] Kreisel J., Pignard S., Vincent H., Sénateur J. P. & Lucazeau G.. Raman study of BaFe_12_O_19_ thin films. Appl. Phys. Lett. 73, 1194–1196 (1998).

[b42] Kreisel J., Lucazeau G. & Vincent H. Raman study of substituted barium ferrite single crystals, BaFe_12-2x_Me_x_Co_x_O_19_ (Me=Ir, Ti). J. Raman Spectrosc. 30, 115–120 (1999).

[b43] El Marssi M., Le Marrec F., Lukyanchuk I. A. & Karkut M. G. Ferroelectric transition in an epitaxial barium titanate thin film: Raman spectroscopy and x-ray diffraction study. J. Appl. Phys. 94, 3307–3312 (2003).

[b44] Harward I., O’Keevan T., Hutchison A., Zagorodnii V. & Celinski Z. A broadband ferromagnetic resonance Ssectrometer to measure thin films up to 70 GHz. Rev. Sci. Instrum. 82, 095115 (2011). 21974627 10.1063/1.3641319

[b45] Meng S., Yue Z. & Li L. In-plane *c*-axis oriented barium hexaferrite films prepared by magnetron sputtering. Mater. Lett. 86, 92–95 (2012).

[b46] Hien N. T. M., Han K., Chen X.-B., Sur J. C. & Yang I.-S. Raman scattering studies of spin-waves in hexagonal BaFe_12_O_19_. J. Raman Spectrosc. 43, 2020–2024 (2012).

[b47] Davydov V. Y. . Raman and photoluminescence studies of biaxial strain in GaN epitaxial layers grown on 6H–SiC. J. Appl. Phys. 82, 5097–5102 (1997).

[b48] Chen H. . Crosshatching on a SiGe film grown on a Si(001) substrate studied by Raman mapping and atomic force microscopy. Phys. Rev. B: Condens. Matter Mater. Phys. 65, 233303 (2002).

[b49] Vasu K., Sreedhara M. B., Ghatak J. & Rao C. N. Atomic layer deposition of *p*-type epitaxial thin films of undoped and N-doped anatase TiO_2_. ACS Appl. Mater. Interfaces 8, 7897–7901 (2016). 26963716 10.1021/acsami.6b00628

[b50] Narayan J. & Larson B. C. Domain epitaxy: A unified paradigm for thin film growth. J. Appl. Phys. 93, 278–285 (2003).

[b51] Akbashev A. R., Plokhikh A. V., Barbash D., Lofland S. E. & Spanier J. E. Crystallization engineering as a route to epitaxial strain control. APL Materials 3, 106102 (2015).

[b52] Smit J. & Wijn H. P. J. Ferrites: Physical Properties of Ferrimagnetic Oxides in Relation to Their Technical Applications. (Wiley, 1959).

[b53] Shinde S. R. . Effect of lattice mismatch strains on the structural and magnetic properties of barium ferrite films. Appl. Phys. Lett. 72, 3443–3445 (1998).

[b54] Dorsey P. C. . Coefficients of thermal expansion for barium hexaferrite. J. Appl. Phys. 79, 3517–3520 (1996).

[b55] Yuan L., Yue Z., Meng S. & Li L. High-frequency ferromagnetic resonance of Co nanowire arrays. Phys. Status Solidi A 211, 1828–1833 (2014).

[b56] Kittel C. On the theory of ferromagnetic resonance absorption. Phys. Rev. 73, 155–161 (1948).

[b57] Vittoria C. Magnetics, Dielectrics, and Wave Propagation with MATLAB^®^ Codes. (CRC Press, 2011).

