# Supplementary material for: Epitaxially grown BaM hexaferrite films having uniaxial axis in the film plane for self-biased devices
Source: Sci Rep. 2017 Mar 9;7:44193. doi: 10.1038/srep44193 (PMC5343456; doi:10.1038/srep44193)
Supplement: Supplementary Information [file srep44193-s1.pdf]

Supplementary Information

Epitaxially grown BaM hexaferrite films having uniaxial axis in the film plane for self-biased devices

Xiaozhi Zhang[1], Siqin Meng[1], Dongsheng Song[2], Yao Zhang[1], Zhenxing Yue[1,*] and Vincent G. Harris[3]

[1]State Key Laboratory of New Ceramics and Fine Processing, School of Materials Science and Engineering, Tsinghua University, Beijing 100084, China

[2]National Center for Electron Microscopy in Beijing, Key Laboratory of Advanced Materials (MOE), School of Materials Science and Engineering, Tsinghua University, Beijing 100084, China

[3]Center for Microwave Magnetic Materials and Integrated Circuits, and the Department of Electrical and Computer Engineering, Northeastern University, Boston, Massachusetts 02115, USA

*Correspondence should be addressed to Z. Y. (email: yuezhx@mail.tsinghua.edu.cn)

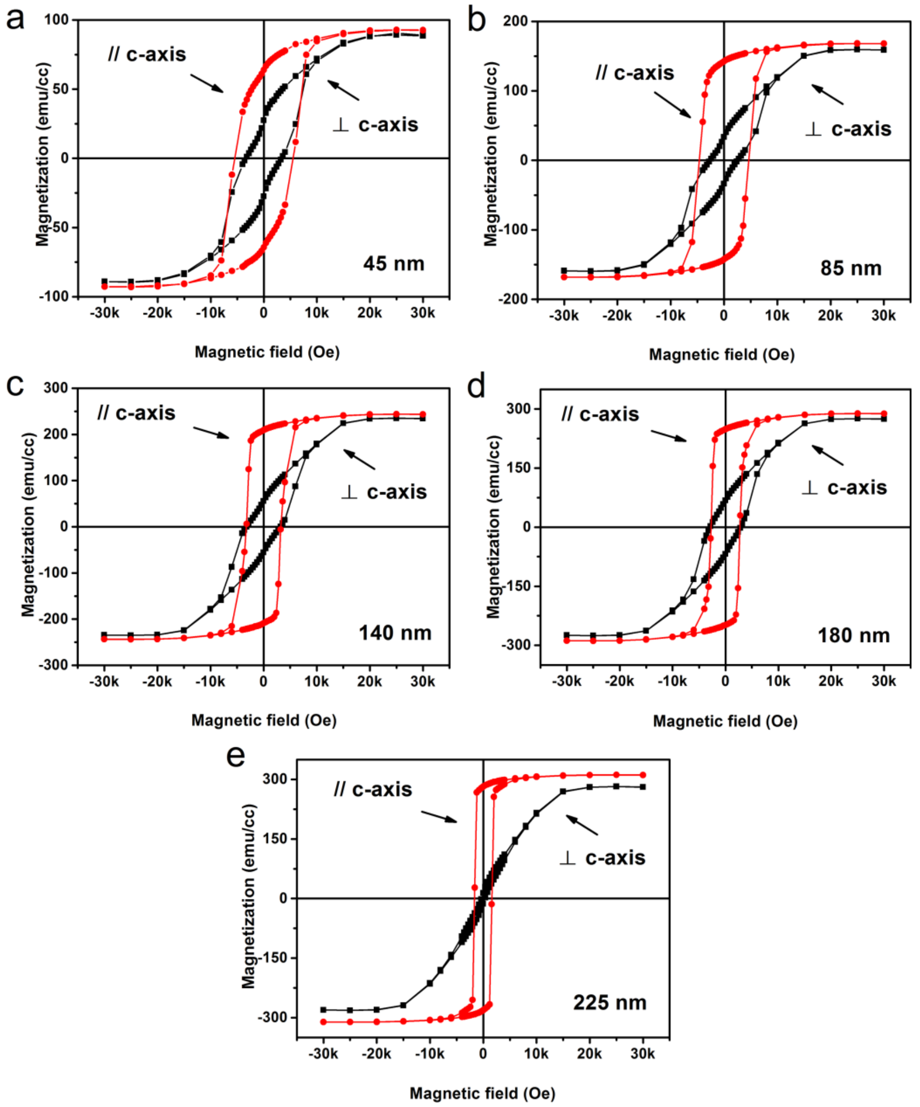

Figure S1. Magnetic hysteresis curves of monolayer BaM films with different thickness, with the loops measured with the magnetic field along the in-plane easy and hard axes of BaM, respectively.

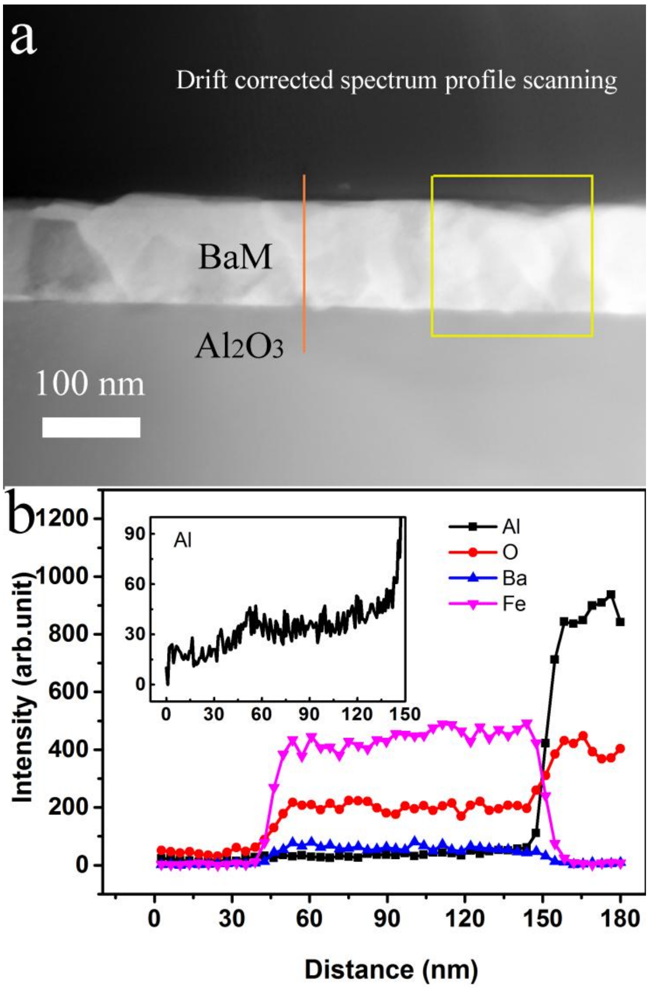

Figure S2. Elemental scan profile of the 130-nm-thick BaM film. The vertical line in (a) denotes the regions where EDS is taken as shown in (b). The yellow rectangular in (a) is the area for drift correction during the experiments. Inset in (b) is the magnification of the concentration of element Al, indicating the diffusion of Al from sapphire substrate to BaM film sample for a long distance.

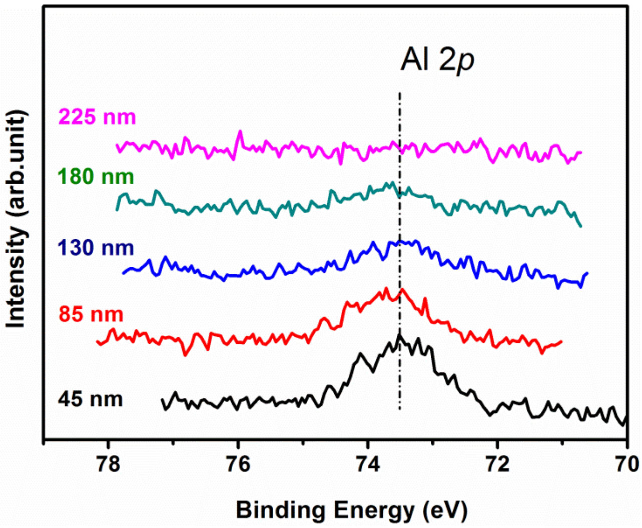

Figure S3. High-resolution scan X-ray photoelectron spectra of Al 2*p* for the BaM film samples with different thickness. Note that the detection depth of XPS is only ~ 10 nm, and the signals should be attributed to the films rather than the substrates. The appearance of aluminum peaks substantiates the diffusion of aluminum from Al_2O_3 substrates to the films, and the intensity decreases with the increasing film thickness. For the 225-nm-thick BaM film, the Al 2*p* peak almost disappears, indicating the diffused Al did not reach to the top of this thick film.
